# Headache as a symptom of Multiple Sclerosis

**DOI:** 10.1186/1129-2377-14-S1-P171

**Published:** 2013-02-21

**Authors:** T Kalamatas, N Protopapas, E Vasiliadis, P Karanasiou, K Karageorgiou

**Affiliations:** 1 GNA G.Gennimatas, Greece

## Introduction

Our aim was to investigate the prevalence of headache in Multiple Sclerosis (MS). We studied data from our department’s day clinic files of MS patients. The associations between headache characteristics and clinical features of MS were investigated.

## Purpose/background/objectives

Our main goal is to study the comorbidity of headache and MS. We investigated possible correlations between MS clinical course and headache characteristics. Headache is a common complaint between Multiple Sclerosis (MS) patients. It has been found that headache prevalence during MS is above 50 % and this is different from the percentage of the general population. There are many studies that show comorbidity of headache and MS (56.2% 2).

## Methods

We retrospectively studied demographics data from MS patients of our department's day clinic (sex, age, duration of MS, presence of headache, type of headache, number of headache attacks per year).

## Results

From 144 patients, 75 (52%) presented with symptoms of headache. 69.2% were female and 30.8% were male. 25% of the patients had headache before MS diagnosis and migraine was a symptom of 61.5% of the above mentioned patients. No statistical significant associations could be determined between duration of MS disease and the frequency, strength and duration of headache.

## Conclusion

Headache with or without other neurological signs could be coexists with MS in greater frequency than the healthy population. The above outcome could be explained by some common underlining biological mechanisms. Further studies are needed to clear up the mechanisms of headache co-morbidity with MS.
